# RB acute loss induces centrosome amplification and aneuploidy in murine primary fibroblasts

**DOI:** 10.1186/1476-4598-5-38

**Published:** 2006-09-20

**Authors:** Flora Iovino, Laura Lentini, Angela Amato, Aldo Di Leonardo

**Affiliations:** 1 Department of Cellular and Developmental Biology "A. Monroy", University of Palermo, Italy

## Abstract

**Background:**

Incorrect segregation of whole chromosomes or parts of chromosome leads to aneuploidy commonly observed in cancer. The correct centrosome duplication, assuring assembly of a bipolar mitotic spindle, is essential for chromosome segregation fidelity and preventing aneuploidy. Alteration of p53 and pRb functions by expression of HPV16-E6 and E7 oncoproteins has been associated with centrosome amplification. However, these last findings could be the result of targeting cellular proteins in addition to pRb by HPV16-E7 oncoprotein. To get a more detailed picture on the role of pRb in chromosomal instability and centrosome amplification, we analyzed the effects of the acute loss of retinoblastoma gene function in primary conditional *Rb *deficient mouse embryonic fibroblasts (MEFs). Moreover, since pRb is a transcriptional repressor, microarray analysis was done on pRb-competent and pRb-deficient MEFs to evaluate changes in expression of genes for centrosome homeostasis and for correct mitosis.

**Results:**

Acute loss of pRb induces centrosome amplification and aneuploidy in the vast majority of cells analyzed. A time course analysis shows a decrease of cells with amplified centrosomes after 40 days from the adenoviral infection. At this time only 12% of cells still show amplified centrosomes. Interestingly, cells with pRb constitutive loss show a similar percentage of cells with amplified centrosomes. DNA-Chip analyses in MEFs wt (mock infected) and pRb depleted (Ad-Cre infected) cells reveal differential expression of genes controlling both centrosome duplication and mitotic progression.

**Conclusion:**

Our findings suggest a direct link between pRb status, centrosome amplification and chromosomal instability, and define specific mitotic genes as targets whose gene expression has to be altered to achieve or maintain aneuploidy.

## Background

Aneuploidy is considered a common feature of tumors and early stage carcinomas. Aneuploidy and its underlying cause (CIN) could be considered a prerequisite in tumor initiation/progression and like alterations in signaling pathways it is believed essential for tumorigenesis [1,2]. However, the molecular defects underlying aneuploidy and whether it is a cause or a consequence of the tumor phenotype are not completely clear. At least two possible causes of aneuploidy could be suggested: mutation/alteration in genes encoding spindle checkpoint proteins and defects in genes controlling centrosome numbers.

Some studies suggest that aneuploidy in cancer cells could arise from defects in the mitotic spindle checkpoint. This mitotic checkpoint monitors that in metaphase, all chromosomes are properly aligned and attached to the mitotic spindle before progressing to anaphase. Although in human and murine cells mutations of some of these mitotic genes such as: *hBUB1*, *hSecurin*, *hMAD2 *and *hBUBR1 *induced aneuploidy in dividing diploid cells over several generations, they were rarely mutated in human tumors [3-7]). Recently, it was reported a hereditary mutation of the *hBUBR1 *gene in patients with mosaic variegated aneuploidy (MVA) a rare genetic disease with increased cancer risk [8]. Also, *MAD2 *overexpression was associated with stable inactivation of the retinoblastoma tumor suppressor and consequently E2F-1 activation [9].

Alternatively, aneuploidy could arise from an initial abnormal chromosome complement that in turn triggers chromosome missegregation and genetic instability [1]. Previously, we showed that transient polyploidization caused by DNA re-replication could be a trigger for aneuploidy in human cells [10]. Moreover, after treatments with anti-mitotic drugs both human and murine pRb deficient cells displayed perpetual aneuploidy and amplified centrosomes [11] suggesting that deregulation of cell cycle progression and alteration in centrosome numbers could be implicated in chromosomal instability.

Loss of centrosome duplication control will often create multipolar spindles that in turn could be responsible for the incorrect segregation of whole chromosomes leading to aneuploidy. Centrosome duplication is coupled to DNA replication and systems of constraints ensure that centrosome duplication occurs during S phase once and only once. In particular, the activation of cyclin E-Cdk2 (cyclin-dependent kinase 2) complex at the G1/S phase transition of the cell cycle allows both DNA replication and centrosome duplication to proceed. [12]. Centrosome amplification was associated with genetic instability in prostate [13] and breast cancer [14] as well as in preinvasive cancer lesions [15]. The observation made in human keratinocytes that centrosome amplification occurs following expression of the HPV16 E6 and E7 oncoproteins [16] suggests a role in this process for the tumor-suppressors p53 and pRb. Also, centrosome amplification induced by a transient G1/S arrest because of the presence of Hydroxyurea, generated aneuploidy both in human and MEFs with pRb dysfunction [11]. However, it is still controversial whether p53 loss/inactivation plays a direct role in centrosome amplification [17] and chromosome instability [18]. Indeed, recently it has been reported that p53 deficient cells need cyclin E over-expression, a target of E2F transcription factor, to induce chromosome instability and centrosome amplification [19]. This suggests that pRb dysfunction, resulting in activation of the E2F family transcription factors, could be crucial in centrosome amplification and chromosome instability.

To get additional clues on the role of pRb in *CIN *we analyzed the effects of acute loss of retinoblastoma gene function on centrosomes and aneuploidy in primary conditional *Rb *deficient Mouse Embryonic Fibroblasts (MEFs). This conditional knockout system permits us to assess the effects of acute loss of the retinoblastoma gene by the Cre-lox-mediated excision of Rb's exon 19. In addition by DNA microarray analysis of these pRb deficient cells we found altered expression of mitotic genes that could account for chromosomal instability associated with centrosome alterations. Our results suggest a direct link between pRb status, centrosome amplification and altered expression of genes necessary for the correct mitosis progression in triggering aneuploidy.

## Results

### Centrosome amplification after acute loss of pRb in primary murine fibroblasts

**Figure 1:**
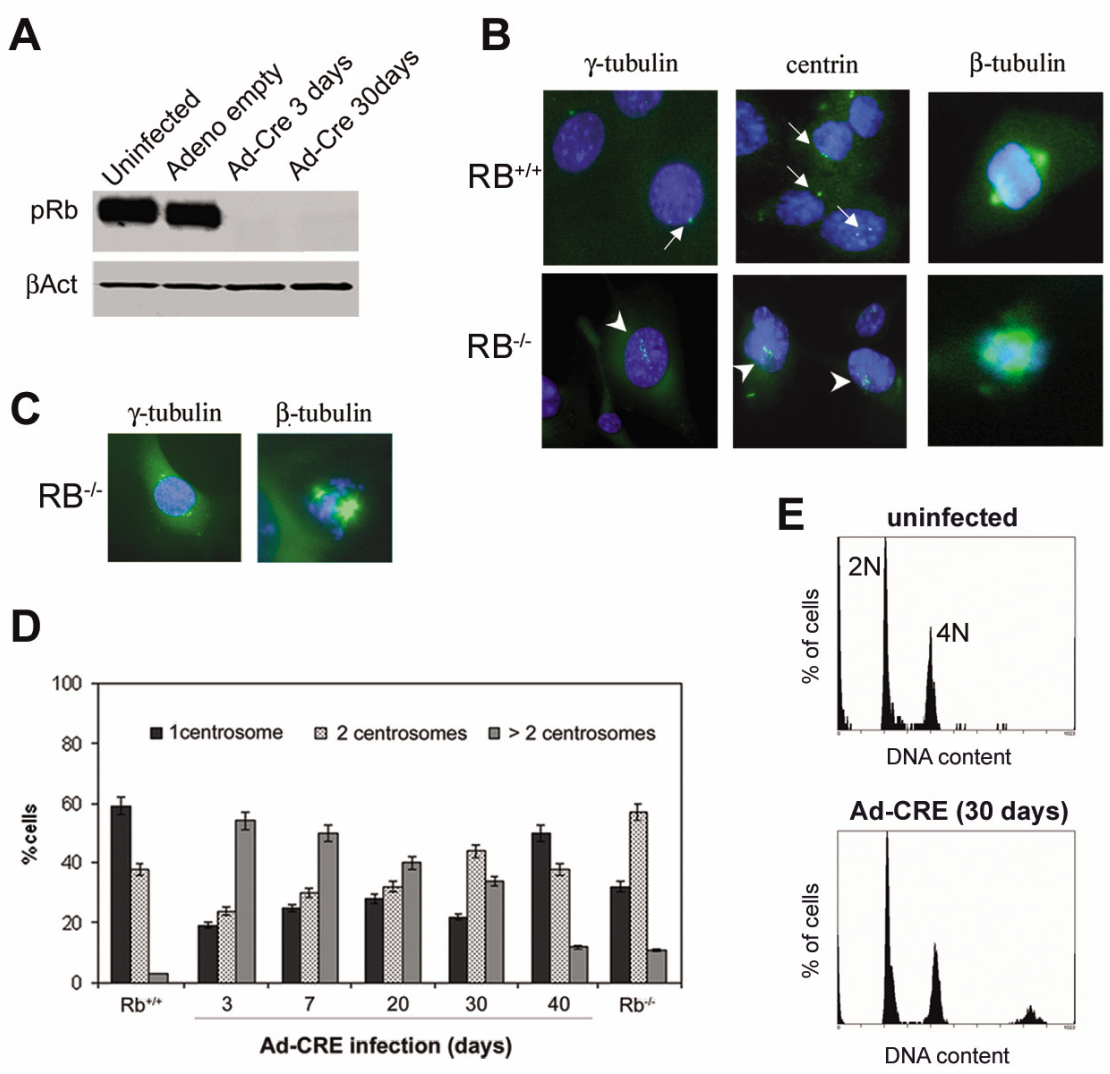
**Centrosome amplification in MEFs after pRb acute loss**. (**A**) Western blot analysis showing the level of pRb expression after 3 and 30 days from the adenoviral infection: Rb^LoxP/LoxP ^MEF uninfected (*lane *1), infected with the empty adenoviral vector (*lane *2) and with the Ad-Cre at 3 (*lane *3) and 30 days (*lane *4). (**B**) Control MEFs (Rb^+/+^) showed 1 or 2 centrosomes, on the contrary MEFs acutely depleted of pRb (Rb^-/-^) showed the presence of supernumerary centrosomes (arrowheads). The number of centrosomes per cell was scored by microscope. Centrosomes were revealed by γ˜
 MathType@MTEF@5@5@+=feaafiart1ev1aaatCvAUfKttLearuWrP9MDH5MBPbIqV92AaeXatLxBI9gBaebbnrfifHhDYfgasaacH8akY=wiFfYdH8Gipec8Eeeu0xXdbba9frFj0=OqFfea0dXdd9vqai=hGuQ8kuc9pgc9s8qqaq=dirpe0xb9q8qiLsFr0=vr0=vr0dc8meaabaqaciaacaGaaeqabaqabeGadaaakeaacuaHZoWzgaacaaaa@2E62@ tubulin and centrin detection (green) and nuclei were stained with DAPI (blue). (**C**) Presence of coalescent centrosomes forming pseudo-bipolar spindles in pRb deficient MEFs as shown by β- and γ-tubulin detection. (**D**) Time course analysis showing a decrease of cells with amplified centrosomes at 40 days from adenoviral infection. Histograms show the percentage of cells with 1, 2 or more than two centrosomes. An average of 100 cells were analyzed for each time point. Each bar indicates mean + s.e. of at least three independent experiments. (**E**) Representative FACScan profiles of cell cycle distribution after 30 days from the adenoviral infection in primary murine fibroblasts.

To evaluate the effects of Rb acute loss on centrosomes we used Mouse Embryonic Fibroblasts (MEFs) carrying conditional Rb alleles (*Rb*^*LoxP*/*LoxP*^) in which two LoxP sites are inserted into the introns surrounding exon 19 of the *Rb *gene [34]. Exponentially growing MEFs (MEF *Rb*^*LoxP*/*LoxP*^) were infected with adenoviruses expressing the Cre recombinase (Ad-Cre) in order to generate cells with truncated pRb (*Rb*^-/-^), by excision of exon 19, that are functionally equivalent to *Rb null *cells. As a control *Rb*^*LoxP*/*LoxP *^MEFs were infected with adenoviruses that did not express the Cre transgene (pRb proficient, indicated as *Ad-empty *MEFs). Western-blot experiments (Figure 1A) showed that in *Rb*^*LoxP*/*LoxP *^MEFs the pRb protein was no longer detectable as soon as 3 days after infection with Ad-Cre in comparison to both uninfected cells and cells infected with adenoviruses that did not express the Cre recombinase. Acute loss of pRb (3 days) in these murine cells resulted in high percentage of cells (55 %) with centrosome amplification as seen by immunocytochemistry against γ-tubulin (Figure 1B). Actual centrosome reduplication was confirmed by immunostaining of centrin (Figure 1B). In addition detection of β-tubulin showed the presence of altered mitotic spindles in cells acutely depleted of pRb (Figure 1B). A time course analysis of the cells acutely depleted of pRb showed (Figure 1D) that the number of cells with amplified centrosomes decreased over time and after 40 days from the transfection only 12% of cells still showed amplified centrosomes. Cells with pRb constitutive loss showed a similar number (10%) of cells with amplified centrosomes. These results are similar to those previously reported in p53^-/- ^MEFs that have about 30% of cells with amplified centrosomes at early passages but after prolonged culture almost of the cells showed a normal centrosome complement (clonal adaptation) [20]. FACScan analyses after Ad-Cre infection showed lack of cells with DNA content less than 2N (Figure 1E) suggesting that in these cells apoptosis has not taking place. This is consistent with previous findings that in murine cells pRb abrogation doesn't induce apoptosis [21].

These results underline a different effect of acute versus chronic loss of pRb and that the majority of cells with amplified centrosomes are not maintained during growth in culture most likely because of chromosomal instability triggered by amplified centrosomes. We also scored a fraction of pRb depleted cells showing clusters of multiple centrosomes at each spindle pole (Figure 1C) that could be a safeguard mechanism by which bipolarity of the spindle is maintained and the cells are unlikely to acquire extensive chromosomal alterations.

### Acute loss of pRb in primary murine fibroblasts is associated with aneuploidy

**Figure 2:**
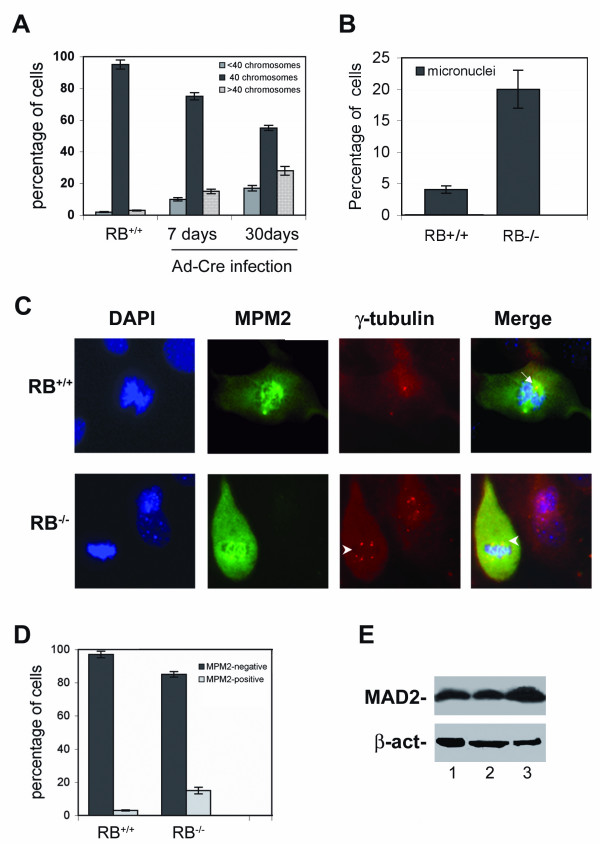
**Acute loss of Rb leads to chromosome instability and increases MPM2 positive cells**. (**A**) pRb deficient MEFs show increased aneuploid metaphases both hypodiploid (<40 chromosomes) and hyperdiploid (>40 chromosomes). Each bar indicates mean + s.e. of three independent experiments. (**B**) pRb deficient MEFs have increased percentage of cells with micronuclei. An average of 100 cells were analyzed for each time point, each bar represents mean + s.e. of two experiments. (**C**) MEFs acutely depleted of pRb (Rb-/-) have increased number of mitotic cells with multiple centrosomes (arrowheads). Mitotic cells and centrosomes were revealed by using primary antibodies against MPM2 (Mitotic Protein Monoclonal) and γ-tubulin, that were detected by FITC (green) and TRITC (red) conjugated secondary antibody respectively. (**D**) Histograms indicating the percentage of MPM2 positive cells in pRb competent and deficient MEFs. An average of 100 cells were analyzed for each time point, each bar represents mean + s.e. of at least two experiments. (**E**) Western blot analysis showing the amount of MAD2 protein after 3 days from the adenoviral infection: Rb^LoxP/LoxP ^MEF uninfected (*lane *1), infected with the empty adenoviral vector (*lane *2) and with the Ad-Cre vector (*lane *3).

Centrosome amplification observed after acute loss of pRb in MEFs might be responsible for chromosomal missegregation and successive aneuploidy. To this aim we examined whether MEFs with acute depletion of pRb had acquired chromosomal instability by carrying out conventional cytogenetic analyses. Alterations in the normal number of chromosomes were frequently observed in MEFs acutely depleted of pRb but not in their wild type parental counterpart. After 7 days from Ad-Cre infection 30% of scored metaphases were aneuploid, both hypodiploid and hyperdiploid (Figure 2A). The percentage of aneuploid metaphases increased to 45–50% after 30 days from Ad-Cre infection suggesting the occurrence of perpetual aneuploidy in these cells. In addition, microscopic examination at 72 hours revealed that these *Rb *'knockout' cells showed a significant increase in the percentage (20%) of cells with micronuclei (Figure 2B) as well as lobulated nuclei. The presence of micronuclei reveals segregation defects and it may explain the generation of hypodiploid mitoses after pRb acute loss. FACScan analyses of wild type and of pRb acutely depleted MEFs (Figure 1E) showed a similar distribution of cells in the G1/S and G2/M phases suggesting that loss of pRb did not influence the cell cycle progression. However, specific evaluation of mitotic cells after 72 hours from AdCre infection by MPM2 staining (Figure 2C), an antibody that recognizes mitotic proteins, revealed a higher number of positive (mitotic) cells (15%) in *Rb *depleted MEFs in comparison to wild type MEFs (Figure 2D). The increased mitotic index suggests that the duration of mitosis in these cells could be prolonged by activation of the mitotic checkpoint as suggested by MAD2 over-expression in these cells (Figure 2E).

### Presence of significant changes in gene expression in cells acutely depleted of pRb

The results above suggest that pRb acute loss in primary murine fibroblasts can both induce centrosome amplification and chromosomal instability (aneuploidy). In order to better understand whether loss of pRb affects expression levels of specific genes underlying centrosome amplification and aneuploidy, we used microarray analysis to identify differentially expressed genes in cells wherein pRb was acutely depleted in respect to gene expression of control cells.

**Figure 3:**
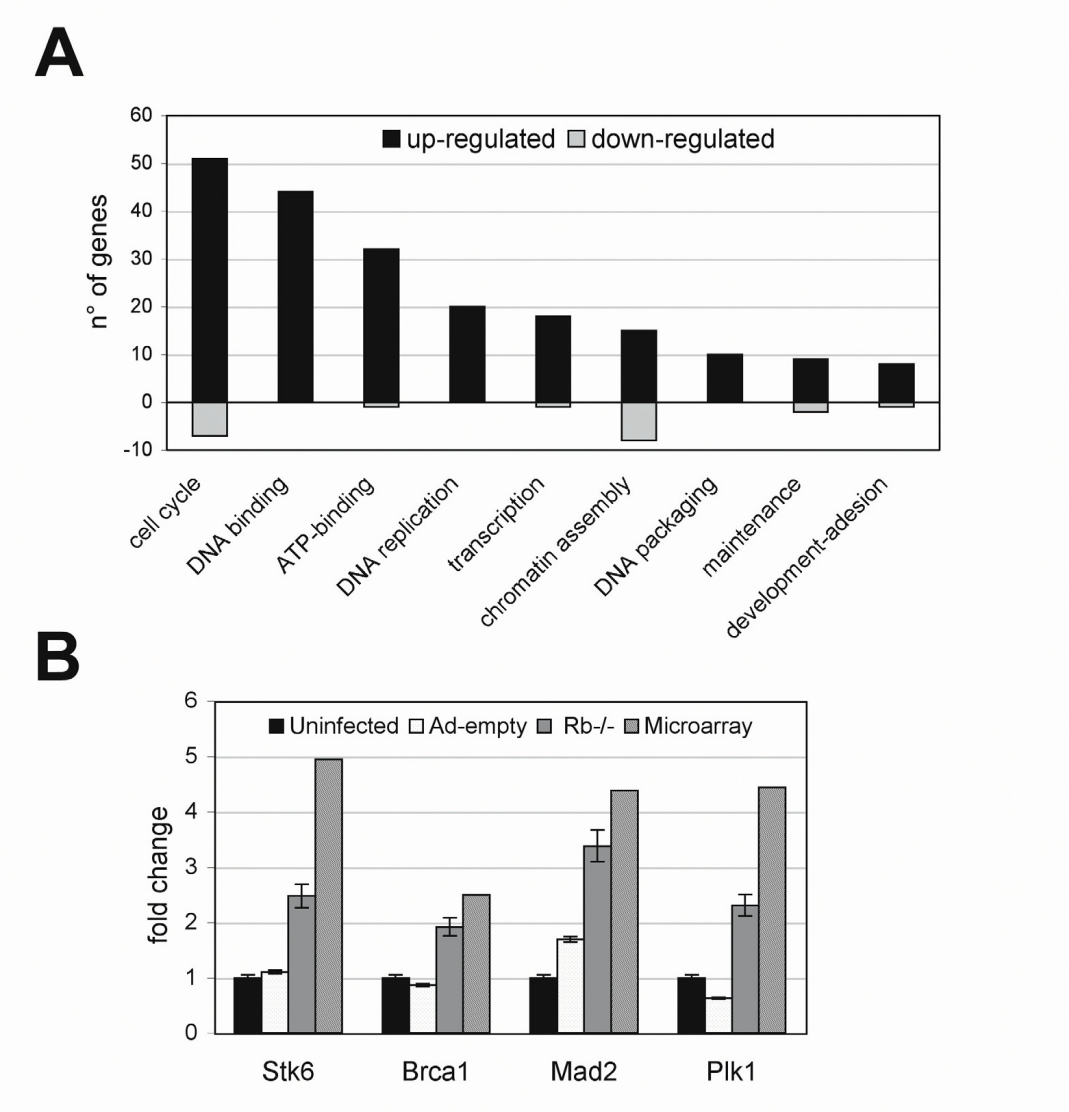
**Functional hierarchies of transcripts**. (A) Overall view of transcript changes. Note that because increased/decreased genes may appear multiple times within these hierarchies, the number of genes is relative, not absolute. (B) Comparison of expression levels of selected genes between real-time RT-PCR data in MEFs: uninfected (black bars), infected with the empty adenoviral vector (white bars) and infected with Ad-Cre (grey bars) and Affymetrix array data (downward diagonal bars).

Independent cultures of asynchronously growing age matched MEFs both pRb proficient and deficient were utilized to isolate RNA. This RNA was then subjected to microarray analyses on the Affymetrix GeneChip^® ^MGU-74A Array platform. The data of the different arrays were compared by means of normalization techniques and clustered to group similar arrays. Differentially expressed genes (DEG) were selected according to a significance level and clustered to group genes with similar expression profiles across the samples. DEGs were then functionally characterized with several databases, and annotation terms were ranked based on their statistical relevance. Finally, a correspondence analysis was performed aimed at highlighting the most relevant genes for each experimental condition. Only the statistically most significant annotation terms were selected (p Value ≤ 1e-^3^) and a minimum statistical value of 2 was used to narrow the list of genes modified by pRb loss. From these analyses we identified 231 individual Affymetrix entries: 186 upregulated and 45 down-regulated [see Additional file 1]. The GO (Gene Ontology) pathway categories showed that the majority of up-regulated transcripts belonged to cell cycle, DNA replication and chromatin assembly. Whereas transcripts with reduced levels were principally associated with cytoskeleton and basal metabolism (Figure 3). As expected many of the up-regulated genes were known E2F targets [see Additional file 1] in particular in the DNA replication category MCM proteins were strongly upregulated by pRb loss. Additional genes regulating the DNA replication complex were also upregulated, including Cdk2 and cyclin-E that phosphorylate members of the replication complex and are necessary for centrosome duplication. These extensive alterations in the DNA replication pathway reflect a general induction of DNA synthesis. Another interesting observation was the up-regulation of cdc2 and cyclin-B, partners of the kinase complex required for progression into mitosis. In addition pRb loss upregulated other genes involved in mitosis progression, centrosome homeostasis and mitotic checkpoint such as Plk1, Brca1 and Stk6 (Aurka), Nek2, Stathmin 1, Ckap2, Pttg1 (Securin), Cdc20, Mad2l1 (Table 1). Most of these genes are considered critical regulators of cell cycle progression and centrosome duplication that exhibit aberrant expression in multiple tumors. Our microarray results clearly suggest that the expression differences observed in cells acutely depleted of pRb could account for the phenotype observed. In order to confirm these results we chose a subset of genes from the mitotic group and performed quantitative Real time RT-PCR analysis to assess whether gene expression levels resulting from microarray analysis were an accurate picture of the transcription taking place. Graphic representation of Real time RT-PCR showed that expression levels were very close to those obtained via microarray (Figure 3B). Although the fold change values between groups may differ, transcripts found to be increased in microarrays were found to be increased in Real time RT-PCR studies.

**Table 1:** Changes in transcript levels of genes associated with mitotic progression after pRb acute loss

**Symbol**	**Stat. Value**	**Description**	**Function**
Stmn1	11,1	stathmin 1	microtubule depolymerization mitotic spindle assembly
Ckap2	8,74	cytoskeleton associated protein 2	microtubule stabilization
Nusap1	8,45	nucleolar and spindle associated protein 1	positive regulation of mitosis
Prc1	7,06	protein regulator of cytokinesis 1	mitotic spindle midzone associated-Cdk dependent
Ccnf	6,76	cyclin F	cell cycle, mitosis
Ccnb2	6,57	cyclin B2	regulation of cell cycle
Anln	6,18	anillin, actin binding protein (scraps homolog, Drosophila)	cytokinesis
Cdc20	6,14	cell division cycle 20 homolog (S. cerevisiae)	cytokinesis
Smc2l1	6,11	SMC2 structural maintenance of chromosomes 2-like 1 (yeast)	mitotic chromosome condensation
Ccnb1	5,98	cyclin B1	DNA replication
Smc4l1	5,91	SMC4 structural maintenance of chromosomes 4-like 1 (yeast)	chromosome segregation and organitation, cytokinesis
Plk4	5,61	polo-like kinase 4 (Drosophila)	cell cycle and centrosome homeostasis
Cdc2a	5,45	cell division cycle 2 homolog A (S. pombe)	mitotic G2 checkpoint
Mdm2	5,41	transformed mouse 3T3 cell double minute 2	traversing start control point of mitotic cell cycle
Mki67	5,34	antigen identified by monoclonal antibody Ki 67	meiosis, cell proliferation
Rad21	5,08	RAD21 homolog (S. pombe)	cytokinesis, DNA repair, apoptosis, mitosis
Cdk2	5,01	cyclin-dependent kinase 2	cytokinesis, cell cycle, mitosis
Stk6	4,95	aurora kinase A	cell cycle and centrosome homeostasis
Anapc5	4,81	anaphase-promoting complex subunit 5	cytokinesis, ubiquitin cycle, cell cycle, mitosis
Pttg1	4,54	pituitary tumor-transforming (securin)	cell cycle, chromosome segregation, mitosis
Plk1	4,44	polo-like kinase 1 (Drosophila)	cell cycle and centrosome homeostasis
Mad2l1	4,38	MAD2 (mitotic arrest deficient, homolog)-like 1 (yeast)	mitotic sister chromatid segregation, cytokinesis, mitotic spindle checkpoint
Cks2	4,15	CDC28 protein kinase regulatory subunit 2	cytokinesis, cell cycle
Rbbp4	4,11	retinoblastoma binding protein 4	transcription
Ccna2	3,94	cyclin A2	regulation of cell cycle, cytokinesis
Nek2	3,7	NIMA (never in mitosis gene a)-related expressed kinase 2	mitotic sister chromatid segregation, cytokinesis, mitosis
Ccne1	3,11	cyclin E1	regulation of progression through cell cycle, DNA replication
Brca1	2,52	Breast Cancer Associated Protein 1	DNA repair, apoptosis, mitosis

Genes are listed by symbol, change in expression level, description and biological function.

## Discussion

Centrosome amplification and chromosome instability (aneuploidy) occur in virtually all solid tumors analyzed and could be considered early features in tumor initiation and progression [22]. Gain or loss of chromosomes (aneuploidy) could generate in a single step multiple changes required for tumor initiation and progression. At least two possible cause of aneuploidy, not mutually exclusive, could be suggested: defects in genes controlling centrosome homeostasis and mutations in genes encoding mitotic checkpoint proteins.

Stable disruption of cell cycle proteins such as pRb, p53, Brca1, altered expression of Aurora kinases, presence of the viral oncoproteins HPV16-E7 and HPV16-E6 have been associated with centrosome amplification. In addition dysfunction of genes involved in the mitotic checkpoint such as Mad2l1 and BUB family members could lead to chromosomal instability (aneuploidy) facilitating tumorigenesis.

In mammalian cells DNA replication and centrosome duplication must be completed successfully before mitosis and are tightly regulated by a control network in which pRb plays an important role. To this regard we showed that centrosome amplification and perpetual aneuploidy occurred in MEFs cells stably devoid of pRb only following a prolonged G1/S arrest because of exposure to hydroxyurea [11]. Similar results were recently reported in normal human cells lacking of p16^ink4A^, a regulator of pRb activity, following hydroxyurea treatment [23]. However, disruption of a checkpoint governed by pRb through inactivation of p16^ink4A ^alone did not lead to centrosome alterations or to chromosomal instability [24].

Here we demonstrate for the first time that also pRb acute loss in conditional deficient *Rb *MEFs resulted in centrosome amplification and aneuploidy highlighting the importance of pRb in the control of genetic stability. These results are consistent with recent findings showing that p53 deficient cells also need cyclin E over-expression, a target of the E2F transcription factor, to induce CIN and centrosome amplification [19]. Moreover, reduction in hCDC4 protein level by siRNA resulted in a high increase in cyclin E levels and was associated with spindle dysfunction and CIN [25]. Conditions that favor disruption of cell cycle transitions may then contribute to centrosome amplification that in turn results in chromosomal instability through assembly of multipolar spindles. Our DNA microarray analysis showed that several pathways were affected in cells acutely depleted of pRb. Although it is difficult to discuss each of them in detail, it is worthwhile to outline the upregulation of genes involved in centrosome homeostasis and mitosis progression. In particular DNA microarray analysis revealed changes in transcripts of mitotic genes not shown previously, in addition to known genes under E2F control that define a pRb null phenotype [26,27]. We found that critical regulators of mitotic progression and centrosome duplication that harbor oncogenic activity and exhibit aberrant expression in multiple tumors such as Plk1, STK6 (Aurora-A), Stathmin 1 and BRCA1 were upregulated after pRb acute loss. Recently, it has been shown that activation of the RB-pathway resulted in the repression of Plk1 promoter activity and that this action was dependent on the SWI/SNF chromatin remodeling complex [28]. Also the increase of Stathmin 1 expression levels could play a role in chromosomal instability in murine cells. In fact recent studies point to Stathmin 1 as an important mitotic regulator highly expressed in many cancers that is under transcriptional control of E2F [29]. Expression of the HPV16-E7 prevented the down-regulation of Stathmin 1 and led to premature exit from G2 after genotoxic stress [30]. Thus, functional complexes containing E2F and RB appear to be essential for repressing expression of critical mitotic regulators. The increase in the mitotic index of cells acutely depleted of pRb suggested the activation of the mitotic checkpoint triggered by MAD2 levels whose increased gene expression was confirmed by Real time RT-PCR. However, the concomitant over-expression of important mitotic regulators such as Aurora-A and cdc20 could allow some cells to override the activated mitotic checkpoint and then result in aneuploidy [31]. Recently, it was reported that in cells stably depleted of pRb via RNA interference by short hairpin RNAs against Rb transcript increased expression of MAD2 led to chromosomal instability [9]. The explanation for this paradoxical effect of MAD2 was that part of the population adapted to the activated checkpoint, and entered the cell cycle as polyploid cells. This could then cause chromosome misdistribution in subsequent mitoses. However, it could be also possible that additional genetic changes arose during the selection (i.e. Aurora-A/cdc20 over-expression) and overrode the activated mitotic checkpoint.

Altogether, our findings strongly suggest that pRb acute loss resulting in centrosome amplification and likely affecting expression of G2/M genes other than G1/S genes could be crucial in CIN.

## Conclusion

Centrosome amplification is a frequent event in almost all types of solid tumors and although it is not completely clear what cellular factors are responsible for chromosome instability associated with initiation of cancer, it is known that the majority of cancers have mutations in components of Rb pathway. The effects of pRb acute loss in primary murine cells seem to link deregulation of cell cycle progression and alteration in centrosome numbers and mitotic genes to induce aneuploidy.

Finally, we hypothesize that the loss of pRb triggered centrosome amplification by altering expression of genes encoding protein for centrosome homeostasis (i.e. cyclin E, Aurora-A, PLK1, Nek2) and induced defects in components of the spindle checkpoint (MAD2) that in turn gave rise to aneuploidy.

## Methods

### Cells and cell culture

Mouse Embryonic Fibroblasts (MEFs) with conditional Rb alleles (Rb^LoxP/LoxP ^MEFs) were infected (MOI: 300, that ensures 90–95% of successful infected fibroblasts) with the empty vector J-pCA13 or with adenoviruses (Ad-Cre) expressing the Cre recombinase under control of the human cytomegalovirus (hCMV) immediate-early promoter to generate MEFs devoid of pRb (Rb^-/-^). All adenoviruses used in this study were replication-deficient (E1 region deleted). Cells were cultured in DMEM supplemented with 10% FBS, 100 units/ml penicillin and 0.1 mg/ml streptomycin (Euroclone Ltd UK).

### Immunoblot analysis

Cells were lysed in SDS/PAGE sample buffer, protein extracts were resuspended in loading buffer (0.125 M Tris-HCl, 4% SDS, 20% Glycerol v/v, 0.2 M dithiothreitol, 0.02% Bromophenol Blue, pH 6.8) and 50 μg of proteins (as determined by the Bradford assay) were loaded per lane on a SDS PAGE gel. After gel electrophoresis proteins were electro transferred onto Immobilon-PVDF membrane (Millipore) blocked in 5% (w/v) no-fat milk in TBST buffer (10 mM Tris pH8.0, 150 mM NaCl, 0.1% Tween 20) at room temperature and incubated overnight at 4°C with the primary antibody. After washes with TBST buffer the blot was incubated in horseradish peroxidase-conjugated secondary antibody (Santa Cruz Biotechnology) diluted 1:2000 for 1 hour RT. To detect amount of pRb and MAD2 proteins blots were probed with mouse monoclonal antibodies anti pRb (554136, Becton Dickinson) and anti MAD2 (Santa Cruz Biotechnology) respectively as recommended by manufacturer. Equal loading of proteins was evaluated by probing the blots with a mouse monoclonal antibody β-actin (A53-16 Sigma). Blots were developed with chemiluminescent reagent (SuperSignalWest Pico, Pierce Rockford, IL) and exposed to CL-Xposure film (Pierce Rockford, IL) for 1 to 5 min.

### Determination of Ploidy

Asynchronous cells were treated with 0.2 μg/ml colcemid (Demecolcine, Sigma, St. Louis, MO) for 4 hours. Cells were harvested by trypsinization, swollen in 75 mM KCl at 37°C, fixed with 3:1 methanol/acetic acid (v/v), and dropped onto clean, ice-cold glass microscope slides. The slides were air-dried and stained with 3% Giemsa in phosphate-buffered saline for 10 min. Chromosome numbers were evaluated using a Zeiss Axioskop microscope under a 100 × objective. At least 50 metaphases were analyzed at each time point.

### Immunofluorescence microscopy

To detect centrosomes cells (4 × 10^4^) were grown onto glass coverslips, fixed in methanol at -20°C, permeabilized with 0.1% Triton X (Sigma, St. Louis, MO) and blocked with 0.1% BSA both at room temperature. Then, coverslips were incubated with a mouse monoclonal antibody against γ-tubulin (Sigma, diluted 1:250 in PBS-BSA 0,1%), centrin (a gift of Prof J. Salisbury, Rochester, diluted 1:5000 in PBS-BSA 0,1%) or β-tubulin (Sigma, diluted 1:250 in PBS-BSA 0,1%) overnight at 4°C, washed in PBS and incubated with a FITC-conjugated goat anti-mouse IgG secondary antibody (Sigma, diluted 1:100 in PBS-BSA 0,1%) for 1 hour at 37°C. Nuclei were visualized with 4', 6-Diamidino-2-phenylindole (DAPI) and examined on a Zeiss Axioskop microscope equipped for fluorescence; images were captured with a CCD digital camera (Axiocam, Zeiss) and then transferred to Adobe PhotoShop for printing.

### RNA preparation for microarray analysis

Total RNA was extracted from MEF cultures by RNAeasy Mini kit according to the manufacture's instruction (Qiagen). RNA was isolated from control cultures (MEF Rb^LoxP/LoxP ^infected with empty adenovirus) at two separated times and from test cultures (MEF Rb^LoxP/LoxP ^infected with adenoviruses expressing Cre) at three separated times, for a total of 5 RNA samples for six crosses. Total RNA was used for double stranded c-DNA synthesis using the Superscript Choise System (Invitrogen, Life Technologies) with a (dT)_24_-T7 promoter region (GGCCAGTGAATTGTAATACGACTCACTATAGGGAGGCGG [dT]_24_). After c-DNA clean-up, cRNA labeled with biotinylated ribonucleotides was synthesized with the BioArray High Yield RNA transcript labeling kit (Enzo, distributed by Affymetrix, Inc.). The cRNA was cleaned-up by Gene chip Sample Cleanup Module (Affymetrix) and fragmented by metal-induced hydrolysis and used for hybridization with mouse MGU74A arrays (Affymetrix). C-RNA samples were hybridized to mouse Affymetrix Chip MGU74A as recommended by Affymetrix. Hybridization and first analysis were conducted at Genopolis Consortium (Milan). DNA chips were scanned with the GeneChip scanner and signals were processed by using the GeneChip expression analysis algorithm (GCOS, Affymetrix).

### Microarray analysis

Four quality checks were performed to verify the quality of sample preparation and hybridization. First of all for each sample the percentage of probe sets with Detection call "Absent" (A) over the total number of probe sets was determined, as well as for the probe sets with Detection call "Present" (P). Next for each sample the mean value of probe set called A and P respectively was determined. This step of the overall analysis was performed on raw data, before sample normalization, thus differences reflected bias that will be eliminated with the next step of the analysis. Finally the ratios between the expression values for 3' and 5' end of GAPDH and β-actin transcripts was determined. This is useful to control the degree of sample degradation. In order to make different chips comparable, it was necessary to normalize signal intensities, using the Qspline method, a robust non-linear method for normalization using array signal distribution analysis and cubic splines. All probe sets called "Absent" (A) over all conditions and replicates were removed (5012). The 95^th ^percentile of all the signals of the entire dataset that were flagged with an absent call is determined and used as a threshold to remove all the remaining probe sets whose expression values are always below this value in each sample (3440). Finally 4036 probe sets remain for the next analysis steps. PLGEM was used for the selection of differentially expressed genes [32]. The PAM-clustering method was used to partition the gene expression profiles into k clusters. The optimal number of clusters is automatically determined, based on quality scores obtained from different trials of clustering. Experimental conditions where replicates are available are averaged and the logarithm of the ratio between each exp. cond. and the condition chosen as the baseline are calculated (log Ratios). The similarity of the expression profiles of genes within a cluster suggests a similarity in their regulation. Even for poorly annotated genes (e.g. ESTs) a functional hint can be drawn from the occurrence of known genes within the same cluster. The most representative Gene Ontology (GO) annotations for DEG from each cluster were identified by determining the probability (p Value) of random occurrence of GO terms (functional enrichment). Only the statistically most significant annotation terms are selected (p Value ≤ 1e-3).

### Real time RT-PCR

Some differentially expressed genes were selected for Real time RT-PCR. The selected sequences (provided by Affymetrix) associated with the probe sets located on the array were tested against public databases to confirm the identity of the genes. Once confirmed, primer were designed with Primer Express software (Applied Biosystems) choosing amplicons of approximately 70–100 bp. Total RNA used for synthesis of microarray probes (above) was reverse-transcribed in a final volume of 100 μL using the High Capacity c-DNA Archive kit (Applied Biosystems) for 10 minutes at 25°C and 2 hours at 37°C. For each sample 2 μL of cDNA, corresponding to 100 ng of reverse transcribed RNA, were analyzed by Real time RT-PCR (95°C for 15 sec, 60°C for 60 sec repeated for 40 cycles), in triplicate, using the ABI PRISM 7300 instrument (Applied Biosystems). Real time RT-PCR was done in a final volume of 25 μl comprising 1× Master Mix SYBR Green (Applied Biosystems) and 0,3 μM of forward and reverse primers for: MAD2 (Fwd: 5'-GCCGAGTTTTTCTCATTTGG-3'; Rev 5'-CCGATTCTTCCCACTTTTCA-3') [33], Plk1 (Fwd:5'-CTTGGGTATCAGCTGTGT GACAA-3'; Rev 5'-TCAAGGAATTGGGATGGGAG-3'); Aurka (Fwd:5'-GTTCCC TTCGGTCCGAAAC-3'; Rev 5'-AATCATTTCCGGAGGCTG-3'; β-actin (Fwd:5'-ACCGTGAAAGATGATGACCCAGA-3'; Rev 5'-GAGGCATACAGGGA CAGCACA-3'); BRCA1 (Fwd:5'-CCTTGGCACAGGTGTCCAC-3'; Rev 5' GCCATTGTCCTCTGTCCAGG-3'). Data were analyzed by averaging triplicates C_t _(cycle threshold). Levels of RNA expression were determined by using the SDS software version (Applied Biosystems) according to the 2^-ΔΔct ^method. Levels of RNA expression of selected genes were normalized to the internal control β˜
 MathType@MTEF@5@5@+=feaafiart1ev1aaatCvAUfKttLearuWrP9MDH5MBPbIqV92AaeXatLxBI9gBaebbnrfifHhDYfgasaacH8akY=wiFfYdH8Gipec8Eeeu0xXdbba9frFj0=OqFfea0dXdd9vqai=hGuQ8kuc9pgc9s8qqaq=dirpe0xb9q8qiLsFr0=vr0=vr0dc8meaabaqaciaacaGaaeqabaqabeGadaaakeaacuaHYoGygaacaaaa@2E5C@ actin.

## Competing interests

None

## Authors' contributions

FI carried out adenoviral infections, RNA preparation, expression analysis by microarray, real time PCR, immunocytochemistry, participated in the design of the study and drafted the manuscript. LL carried out cytogenetics studies, and helped to draft the manuscript. AA performed immunoassays and helped to draft the manuscript. ADL conceived of the study, participated in its design and coordination, and drafted the manuscript. All authors read and approved the final manuscript.

## Supplementary Material

Additional File 1 **differentially expressed genes**. Identification of genes both up-regulated and down-regulated by pRb acute loss Click here for file
